# Hand Motor Fatigability Induced by a Simple Isometric Task in Spinal Cord Injury

**DOI:** 10.3390/jcm11175108

**Published:** 2022-08-30

**Authors:** Ana Onate-Figuérez, Vanesa Soto-León, Juan Avendaño-Coy, Laura Mordillo-Mateos, Yolanda A. Pérez-Borrego, Carolina Redondo-Galán, Pablo Arias, Antonio Oliviero

**Affiliations:** 1 FENNSI Group, National Hospital for Paraplegics, SESCAM, 45071 Toledo, Spain; 2 Department of Physiotherapy, Universidad de Castilla La Mancha, 45071 Toledo, Spain; 3 GIFTO Group, Faculty of Physiotherapy and Nursing, Universidad de Castilla La Mancha (UCLM), 45071 Toledo, Spain; 4 Rehabilitation Department, National Hospital for Paraplegics, SESCAM, 45071 Toledo, Spain; 5 Universidade da Coruña, NEUROcom (Neuroscience and Motor Control Group), Department of Physiotherapy, Medicine and Biomedical Sciences-INEF Galicia, 15001 A Coruña, Spain; 6 Biomedical Institute of A Coruña (INIBIC), 15001 A Coruña, Spain; 7 Advanced Rehabilitation Unit, Hospital Los Madroños, 28690 Brunete, Spain

**Keywords:** fatigue, isometric contractions, spinal cord injury, fatigue severity scale, human

## Abstract

This study aimed: (1) to evaluate the hand motor fatigability in people with spinal cord injury (SCI) and compare it with measurements obtained form an able-bodied population; (2) to compare the hand motor fatigability in people with tetraplegia and in people with paraplegia; and (3) to analyse if motor fatigability is different in people with SCI with and without clinical significant perceived fatigability. Materials and Methods: 96 participants with SCI (40 cervical and 56 thoracolumbar) and 63 able-bodied controls performed a simple hand isometric task to assess motor fatigability. The Fatigue Severity Scale was used for perceived fatigability evaluation. Results: The main results of this study can be summarized as follows: (1) the waning in muscle force (motor fatigability) during a fatiguing task is similar in controls and participants with SCI; (2) the motor fatigability is influenced by the maximal muscle force (measured at the beginning of the task); and (3) the perceived fatigability and the motor fatigability are largely independent in the individuals with SCI. Conclusion: Our findings suggest that the capability to maintain a prolonged effort is preserved in SCI, and this capacity depends on the residual maximal muscle force in people with SCI.

## 1. Introduction

Spinal Cord Injury (SCI) has a great impact on quality of life [1,2], and fatigue is a common secondary symptom [3,4,5,6] that affects 30–50% people living with SCI. A limited number of specific approaches are available for treating fatigue and, moreover, these are often under-mentioned and/or underestimated in medical interviews with people with SCI [5,7,8,9]. 

Fatigue can be generally defined as a subjective lack of physical and/or mental energy that is perceived by the individual to interfere with usual and desired activities (or work capacity) [10,11]. The level of fatigability experienced by an individual can be estimated (perceived fatigability) or measured directly (objective fatigability) [12]. In this paper, we will refer to “motor fatigability” as a measure of “objective fatigability” (i.e., magnitude of the change in a performance metric after completing a prescribed task), and to “perceived fatigability” as a subjective estimate of past or future work capacity [12]. These measurements are not independent as objective fatigability determines and are determined by the perceived fatigability [11]. 

Attempts to delimit fatigue typically define its acute expression as a change in performance of a task over time, due to both physiological and psychological factors [13]. Fatigue may also be classified as acute or chronic (duration >6 months) [14], generally, acute muscle fatigue is triggered by overstrain in healthy subjects and is mediated by the nervous system, metabolic and muscular factors [15,16,17,18,19], and a typical expression of motor fatigue is the reduction in the maximal voluntary contraction (MVC) muscle force, which recovers after some rest [15,16,17,18,19].

On the other hand, chronic fatigue lacks an identifiable triggering event and is persistent, and this expression of fatigue is more characteristic in pathologies. Muscle fatigue is also usually classified as central or peripheral fatigue depending on the part of the nervous system principally involved. Muscle fatigue is considered to be “central” when it is generated proximal to the neuromuscular junction and as peripheral fatigue when it is generated distally (also defined as muscular intrinsic fatigue) [14,17,20]. 

In pathologies, fatigue can be associated to several factors, such as anxiety, stress, depression, pain and medication [3,9,20,21,22,23,24,25]. All these factors may be simultaneously present in people with SCI [3,20,21,22,23,24,25]. Muscle fatigue may also arise after SCI because the central nervous system fails to adequately drive the spinal motoneurons [19,22,23]. 

The main aim of this study was to measure the motor fatigability of hand muscles (we will refer to this as hand motor fatigability) in people with SCI. The motor fatigability was measured evaluating the temporal course of a sustained isometric task [26] and compared it with measurements obtained form an able-bodied population. Moreover, as elevated perceived fatigability has been reported in people with SCI [3,4,5,6,22,27,28,29,30], we analysed if motor fatigability is related to the perceived fatigability (measured using the Fatigue Severity Scale (FSS) [6]). We compared the hand motor fatigability in people with tetraplegia and in people with paraplegia (in which the hand should be not affected) [31]. In this way, we can investigate how hand motor connections are important to determine motor fatigability in people with SCI. 

## 2. Methods

### 2.1. Participants

The individuals included in this study were patients with SCI and able-bodied controls recruited at the “National Hospital for Paraplegics” located in Toledo (Spain). Able-bodied controls were hospital workers and family members or friends of people with SCI. The able-bodied controls were used to compare their motor performance with the SCI individual performance. All subjects gave their informed consent for inclusion before they participated in the study. The study was conducted in accordance with the Declaration of Helsinki, and the protocol was approved by the Ethics Committee of Toledo Area, Spain (Project identification code: 87-2015). 

Patients’ inclusion criteria were: (1) age > 18 years old; (2) diagnosis of SCI below C1 neurological level without any other severe medical condition; (3) time since injury of more than one month; (4) ability to perform the isometric task proposed, at least with one hand; (5) ability to complete the FSS; and (6) capability to consent participation. Criteria 1, 4 and 6 were also required to participate as able-bodied controls. 

### 2.2. Demographic and Clinical Data

Demographic data concerning sex and age were collected for all participants. 

In the SCI group, we used the international standards for neurological classification of spinal cord injury (ISNCSCI) to collect the clinical data, such as the etiology of the lesion and time since the SCI lesion; the ASIA impairment scale [32] (AIS: A to E); complete or incomplete loss of motor function; and motor score for upper and lower extremities (UEMS and LEMS, respectively score for each one from 0 to 50). The participants were grouped into SCI modalities depending on the neurological level of SCI (cervical or thoracolumbar).

The fatiguing task (see below) was performed with the dominant hand; however, in some people with cervical SCI, this was not the case (due to the cervical neurological lesion, this subgroup of participants preferred to use the non-dominant hand). Thus, we obtained a motor score of the upper extremity the participant used to perform the task (preferred hand) with a possible score from 0 to 25.

Moreover, we collected info about pain with a numeric rating scale (NRS) [33], spasticity with Modified Ashworth Scale (MAS) [34] and depressive mood with a binary assessed (yes or not) from the SCI group. These data were collected for two reasons: (1) they are part of the SCI syndromes; and (2) these symptoms and the medication used to treat them may affect fatigue. 

The FSS, registered before the participants with SCI performed the hand task, was used for perceived fatigability evaluation. This is the most widely used questionnaire to assess fatigue severity in neurological disorders, both in clinical practice and research [2,4,5,24,25,35,36,37]. It was valid for people with SCI [6]. The FSS was administered to all participants and the full nine item completion was required. The items are scored on a 7-point scale, with 1 = strongly disagree and 7 = strongly agree. The criteria used to determine clinically significant fatigue (CSF) was to obtain a score (considering the mean scores from the nine items) greater than or equal to 4 in the FSS [7]. FSS results were obtained from all the participants with SCI. 

### 2.3. Motor Performance: Hand Function and Motor Fatigability

**Figure 1:**
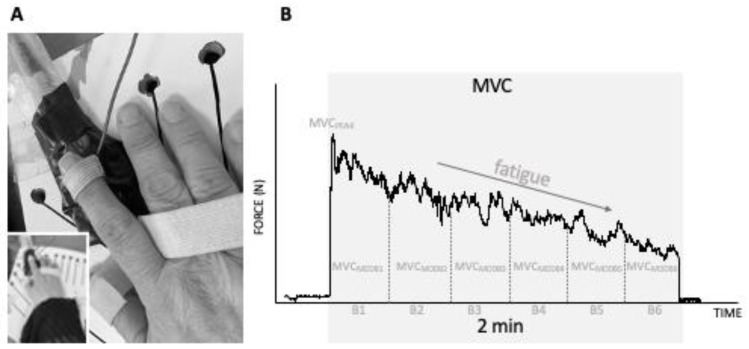
(**A**). The fixation system and hand preparation to execute the isometric task with an adapted dynamometer. (**B**). An example of a two-minute isometric task register. The isometric task is evaluated with the modulus of maximal voluntary contractions (MVC_MOD_) in 20 s periods (B1, B2, B3, B4, B5 and B6). The force measured in Newton (N) decreases along the 2 min and is at the maximum at the beginning of the task (MVC_PEAK_).

After the clinical evaluation and the demographic interview, we performed the hand motor task to evaluate the motor fatigability. An objective evaluation of motor fatigability (muscle fatigue) was performed by testing the decrease in hand MVC force over 2 min (Figure 1). The participants were sitting comfortably with the elbow flexed at 90–100°, with the wrist and forearm secured in a fixation system. 

Participants applied pressure over a thin metal plate located on the force sensor from Biometrics DataLink (Biometrics Ltd., Gwent, UK), and both were attached to the fixation system. The task was performed using the preferred hand. In most of the participants, the preferred hand was the dominant hand; however, in some people with cervical SCI, this was not the case. Participants executed a continuous index finger isometric maximal voluntary contraction (MVC) against the force sensor placed flat on the fixation system. The force direction was “towards” flexion of the first metacarpophalangeal joint. The dynamometer recorded (at 100 Hz) the isometric force exerted during the MVC, during the task. The instructions given to participants before the task was “to press the index finger against the force sensor as hard as you can”, and they also received verbal encouragement during task execution. 

The variables used to describe the motor performance were the maximal voluntary contraction force peak (MVC_PEAK_), the modulus of the maximal voluntary contraction (MVC_MOD_) and the fatigability of the modulus of the maximal voluntary contraction (MVC_MOD_F). These variables were analysed at the beginning and during the task execution. 

The MVC_PEAK_ was defined as the highest peak strength (Newton, N) obtained at any time during the 2 min isometric task. Moreover, the whole force recording (2 min) was analysed by dividing it in 20 s’ consecutive blocks (six blocks in total, namely B1–B6). For each block, the MVC_MOD_ force was quantified, and the data were expressed as Newton per seconds (N·s). Thus, for each variable, we obtained six time points that were included in the statistical analysis to evaluate the temporal decay of the force, i.e., the decay in MVC_MOD_ [38].

Using the same data, we also obtained a simplified variable. Thus, we evaluated the decay over the 2 min as a marker of the fatigability by computing the ratio of the motor output in the last 20 s (MVC_MOD_ calculated in B6) compared with the first 20 s (MVC_MOD_ calculated in B1 of the task. We will refer to this variable as MVC_MOD_ fatigability (MVC_MOD_F = MVC_MODB6_/MVC_MODB1_ × 100). Therefore, a lower MVC_MOD_F indicates higher hand fatigability. These scores are essentially similar to the MVC_MOD_, and we computed it to express motor fatigability in a single parameter.

### 2.4. Statistics

Parametric univariate tests (*t* test) and nonparametric univariate tests (Mann–Whitney *U* test and chi-squared test) were used to compare demographic variables and the clinical data between thoracolumbar and cervical participants with SCI and traumatic and non-traumatic SCI. Sex and age were also compared between participants with SCI and able-bodied. 

Hand motor fatigability (MVC_MOD_F) and the hand muscle force (MVC_MOD_ and MVC_PEAK_) during the isometric task between the participants with cervical SCI, thoracolumbar SCI and the control participants were compared to one-way ANOVA. In the case of significant effects, we used Tukey’s test for post hoc analysis. 

In people with SCI, MVC_MOD_F, MVC_MOD_ and MVC_PEAK_ were also compared after categorizing participants with SCI depending on the presence or not of CSF (FSS ≥ 4). One-way ANOVA was used for this comparison. In addition, we analysed the data using the Bayesian ANOVA methodology to describe how large is the evidence in favour of an effect (both for null and alternative hypothesis). 

A repeated-measure ANOVA design was used with the raw data and MVC_MOD_ normalized data (data were normalized to the first 20 s, with MVC_MODB1_) with BLOCK (B1, B2, B3, B4, B5 and B6) as within-subject’s factors and GROUP (Control, Thoracolumbar or Cervical) as between-subject factors. During ANOVA execution, the degrees of freedom were corrected with Greenhouse–Geisser Coefficients if sphericity could not be assumed. 

In addition, we analysed the data also using the Bayesian Repeated Measures ANOVA to describe how large is the evidence in favour of an effect (both for null and alternative hypothesis). All statistical analyses were performed with the software JASP. The results were considered statistically significant at *p* < 0.05 and the Bayes Factor was described and interpreted according to the article [39]. The “U” in the Bayes factor for post hoc comparisons denotes that it is uncorrected, and the posterior odds have been corrected for multiple testing by fixing to 0.5 the prior probability that the null hypothesis holds across the post hoc tests [40].

## 3. Results

### 3.1. Demographic and Clinical Variables

A total of 96 participants with SCI (37 females; mean age 47.32 ± 17.61 years, range 18–84 years) and 63 able-bodied controls (36 females; mean age 47.7 ± 17.6 years, range 21–77 years) were included in the study. SCI group included more males than females compared to the control group (61.45% vs. 42.85%, *p* = 0.021); age did not differ significantly between SCI and controls *p* = 0.904)

Clinical and demographic data from the participants with cervical SCI (*n* = 40) and thoracolumbar SCI (*n =* 56) are reported in Table 1. There were no substantial differences between traumatic and non-traumatic SCI regarding AIS, neurological level, time since injury, UEMS, LEMS, pain, depressive mood, spasticity and sex. The mean age of non-traumatic SCI was eleven years higher than traumatic (unpaired *t* test, *p* = 0.003).

The mean FSS was 3.1 ± 1.5 and CSF was present in 27.1% of the participants with SCI. The mean FSS was similar in the cervical SCI and the thoracolumbar SCI individuals, and also no significant difference was found when comparing the presence of CSF in both groups (Table 1).

### 3.2. Motor Performance: Hand Function and Motor Fatigability

**Figure 2:**
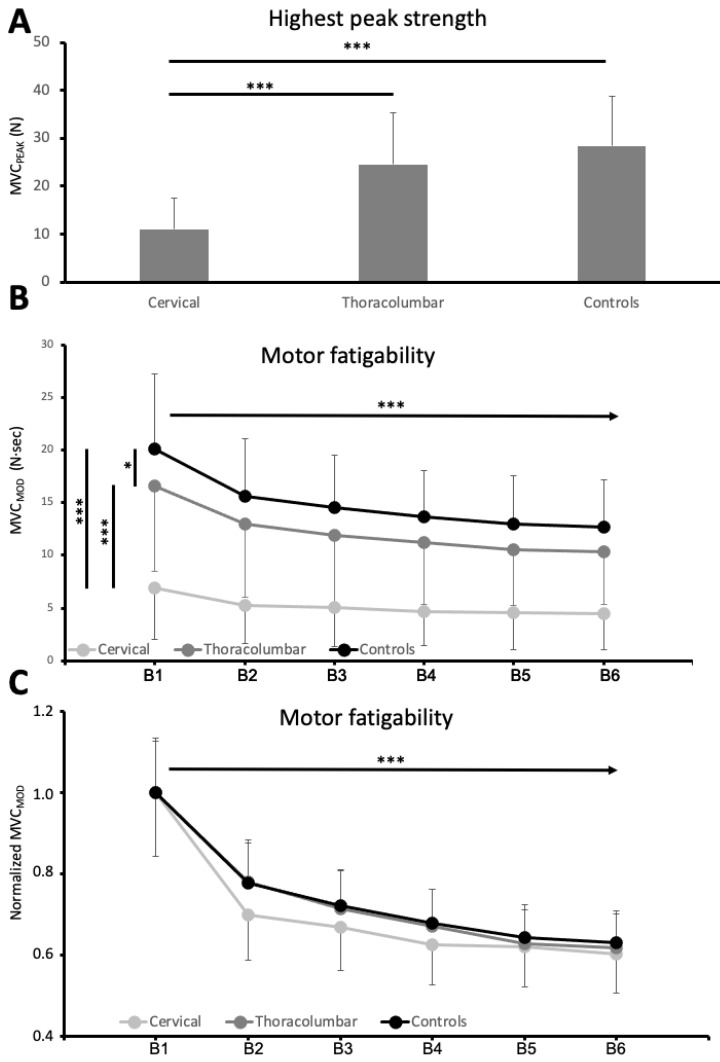
The highest maximal voluntary contractions and motor performance decrease induced by the isometric task. (**A**). The highest scores of force performed during the 120 s of isometric task (MVC_PEAK_) for each group (cervical, thoracolumnar and controls) measured in Newton (N). (**B**). Reduction of muscle force along the 120 s task for each group (cervical, thoracolumnar and controls) measured in Newton·seconds (N·s). The unit in the y-axis represents to the modulus of maximal voluntary contractions MVC_MOD_, and x-axis represents 120 s task in 6 sequential 20 s periods (B1, B2, B3, B4, B5 and B6). (**C**). Normalized Figure 2B respect to the maximal MVC_MOD_ for each group. Asterisks denote statistical significance *** *p* value < 0.001; * *p* < 0.05.

As expected, ANOVA (F_2,156_ = 41.38, *p* < 0.001) revealed that the highest scores of muscle force (MVC_PEAK_) achieved (at any point along the 2 min isometric task, usually at the beginning) was lower in participants with cervical SCI than participants with thoracolumbar SCI and able-bodied participants (Tukey’s test post hoc: cervical SCI vs. thoracolumbar SCI *p* < 0.001; cervical SCI vs. control *p* < 0.001). Participants with thoracolumbar SCI had lower MVC_PEAK_ than controls; however, the differences did not reach significance (Tukey’s test post hoc: control vs. thoracolumbar SCI *p* = 0.076; Figure 2A). 

Bayesian statistics confirmed these observations. The Bayesian ANOVA revealed that groups (cervical SCI, thoracolumbar SCI and controls) had an effect on MVC_PEAK_ with strong evidence (BF_10_ > 100). The post hoc analysis shows strong evidence (BF_10,U_ > 100) that MVC_PEAK_ differed between cervical SCI and thoracolumbar SCI and between cervical SCI and controls. On the other hand, MVC_PEAK_ was different between the control and thoracolumbar SCI with anecdotal evidence (BF_10,U_ = 1.204)

The MVC_MOD_ in the first 20 sec of the task was different between the groups (ANOVA: F_2,156_ = 44.13, *p* < 0.001). MVC_MOD_ was lower in participants with cervical SCI than in participants with thoracolumbar SCI (Tukey’s test post hoc: cervical SCI vs. thoracolumbar SCI, *p* < 0.001) and weaker than in able-bodied controls (Table 2, Tukey’s test post hoc: cervical SCI vs. controls *p* < 0.001, thoracolumbar SCI vs. controls *p* = 0.017). Bayesian statistics confirmed these observations. The Bayesian ANOVA revealed that groups (cervical SCI, thoracolumbar SCI and controls) influenced the MVC_MOD_ with strong evidence (BF_10_ > 100). Post hoc analysis shows strong evidence (BF_10,U_ > 100) that MVC_MOD_ differed between cervical SCI and thoracolumbar SCI and between cervical SCI and controls. The MVC_MOD_ was different between the control and thoracolumbar with moderate evidence (BF_10,U_ = 3.621).

Along the 2 min task, muscle force (i.e., raw MVC_MOD_ at the six sequential 20 s periods) decayed progressively (repeated measures ANOVA, TIME: F_2.1,333,5_ = 140.09, *p* < 0.001). At all time points, the control group produced larger amounts of force than thoracolumbar SCI group, and these group produced more force than cervical SCI group (GROUP: F_2,156_ = 46.76, *p* < 0.001). The way the force dropped with time was different for the three groups (TIMExGROUP: F_10,780_ = 10.04 *p* < 0.001). All these effects had the evidence confirmed by Bayesian Repeated Measures ANOVA (TIMExGROUP: BF_Incl_ > 100; TIME: BF_Incl_ > 100; TIME: BF_Incl_ > 100). These findings are shown in Figure 2B. 

However, most of the differences observed among groups can be explained by the different initial MVC_PEAK_ and MVC_MOD,_ and thus we performed a second analysis with normalized data (expressing force relative to the maximum acquired along the task). MVC_MOD_ progressively decayed up to about 65% of first 20 sec values (repeated measures ANOVA, TIME: F_2.15,336.5_ = 154.3, *p* < 0.001) with strong evidence (Bayesian Repeated Measures ANOVA, TIME: BF_Incl_ > 100). 

Remarkably, the profile of this reduction (hand motor fatigability) did not differ in the three groups (repeated measures ANOVA, GROUP: F_2,156_ = 0.002 *p* = 0.998; TIMExGROUP: F_10,780_ = 0.349 *p* = 0.967). The main effect of GROUP (Bayesian Repeated Measures ANOVA: Group, BF_Incl_ = 0.166) indicates anecdotal evidence that the normalized MVC_MOD_ is the same between groups. On the other hand, the effect of interaction (TIMExGROUP) provides extremely strong evidence (Bayesian Repeated Measures ANOVA: TIMExGROUP, BF_Incl_ < 0.001) that the normalized MVC_MOD_ along the task is the not different among groups. These findings are shown in Figure 2C.

MVC_MOD_F (the change from Block1 to Block6) was similar in the three groups (cervical = 63.71 ± 23.0%; thoracolumbar = 64.67 ± 20.71%; controls = 65.97 ± 19.40%, ANOVA: F_2,156_ = 0.148, *p* = 0.862) with strong evidence confirmed by Bayesian ANOVA (BF_10_ = 0.024). Moreover, MVC_MOD_F was similar in participants with SCI with and without CSF (ANOVA: F_1,94_ = 0.074, *p* = 0.787) with anecdotal evidence observed using Bayesian ANOVA (BF_10_ = 0.245).

## 4. Discussion

The SCI cohort we studied had similar characteristics and perceived fatigability to that reported elsewhere. Our data confirm that people with SCI have elevated levels of perceived fatigue and that they may have frequently clinically significant fatigue (one third of our cohort) [20,21,22,23,24,25]. Moreover, our data suggest that there is no difference between perceived fatigue of tetraplegic and paraplegic people with SCI. In people with cervical SCI, the upper extremity muscles are, at least partially, disconnected from the brain (both efferent and afferent connections), and the spinal motor neurons can be damaged. 

The physiological consequence is that the maximal strength (measured at the beginning of the task we studied) is lower. This agrees with a previous study that reported that maximal voluntary contractions of the flexor carpi radialis showed significantly lower muscle activation in people with SCI compared with in the controls only at the beginning of the task [41]. Our data suggests that muscle force is less in people with cervical SCI (expected) but also slightly less in people with thoracolumbar SCI (not expected). We demonstrated a reduction of the hand motor strength (the maximal strength measured at the beginning of the task is lower compared to controls) in paraplegic individuals. 

We observed similar reduction of the hand motor strength in paraplegic individuals also in other cohort studies (unpublished data). As far as people with thoracolumbar SCI are concerned, that MVC_MOD_ is reduced (without evidence of parallel reduction of MVC_PEAK_) compared to the controls may have two possible, not alternative, explanations: (1) MVC_PEAK_ is also slightly reduced, and modulus calculation increases the statistics sensitivity; (2) the MVC_PEAK_ is similar; however, fatigue occurs faster. This latter explanation would suggest that 2 min of MVC may fatigue only the fast and stronger muscular fibers that are activated at the beginning of the effort. This potential mechanism would be different in people with cervical SCI in which lower muscle activation seems to be more evident at the beginning of the task [41]. 

On the other hand, we compared the hand motor fatigability in people with tetraplegia and in people with paraplegia [31]. In this way, we can attempt to understand how hand motor connections are important to determine motor fatigability in people with SCI. The brain-to-muscle and muscle-to-brain connections are anatomically and functional impaired in people with cervical SCI and only functionally impaired in people with thoracolumbar SCI (data presented here and unpublished data); thus, we expected an incremented hand motor fatigability compared to controls (e.g., more motor fatigability in SCI than in controls and more motor fatigability in cervical than in thoracolumbar SCI).

We confirmed that the exerted forced decay (hand motor fatigability) during an isometric task executed at maximal strength. Our findings provide strong evidence that the force decay during isometric task is similar between participants with SCI and controls [41]. Moreover, we cannot find a difference between cervical and thoracolumbar SCI groups. Similar hand motor fatigability in controls and people with SCI, during a fatiguing task, has been previously reported [42]. Altogether, this data suggests that partial disconnection from the brain (of both efferent and afferent connections) has a greater impact on the muscle strength but not on the motor fatigability. 

The participants with SCI that were able to exert stronger force at the beginning of the task were more prone to become fatigued at the end of the task. On the other hand, we found similar results also in the control population, suggesting that motivation and probably the individual intrinsic capability to exert a stronger force may be more important than the motor system damage. 

Moreover, this suggests that SCI does not affect large and small motor units (MU) in a different way (at least in our sample). If a large MU would be the most affected, the drop in muscle force would be likely shallowed along the 2 min task, which was not the case. If a smaller MU would be the most affected, high levels of muscle force would be less affected; however, the drop in force would be fast, which was not the case in either.

The sustained isometric task we used to test hand motor fatigability (objective measurements of muscle fatigue) does not allow directly providing information about the origin of fatigue (e.g., central or peripheral). Of course, both central and peripheral fatigue will determine the decay of the motor performance. Our data may suggest possible mechanisms to allow participants with SCI, regardless of their severity and neurological level, to keep up with able-bodied controls in their profile of muscle force decay when performing an intense motor task. 

For example, it is likely that the weaker the central drive, the lower requirements at peripheral level and, therefore, a lower development of peripheral fatigue [41]. Lin et al. reported that, in individuals with incomplete SCI, the deficit in the motor brain-to-spine connection (central motor drive) is an important source of muscle weakness and fatigue in the muscle below the level of injury [41]. If so, within our experiment, even in the presence of reduced peripheral fatigue, we should have observed a difference in force decay in individuals with reduced central motor drive (e.g., people with cervical SCI) that we could not observe. 

This difference can be explained by the different motor tasks used. Lin et al. used a repetitive model of isometric contraction, while we used a unique prolonged isometric contraction. It is possible that a single activation of the central motor drive could not produce as much central motor fatigue as a repetitive model (and probably even less peripheral fatigue). Supporting this explanation, fatigue of the motor system is known to be task-dependent [43,44]. For instance, in healthy humans, fatigue produce by maximal isometric contractions appears to have a different impact on excitability of the spinal cord and intracortical M1 circuits compared with maximal rate repetitive movements [19,45], with the latter exerting different effects on muscle force or central drive to the muscle when performed without resistance [46].

The muscle of SCI individuals has different contractility properties [47,48,49]. Can the contractility properties contribute to the lack of motor fatigue after an isometric motor task in people with cervical SCI? In physiological conditions, muscle contractility properties (e.g., slowing) contributes to muscle tension maintenance when motor unit firing rates decay during fatiguing contraction [15]. The muscle slowing in people with cervical SCI may contribute to compensate for the reduced motor central drive. We did not directly evaluate the contractility properties in our sample; therefore, we cannot discard this possibility. 

Whatever the explanation, we hypothesize that the reduced motor output may prevent an excessive fatigability of motor system in SCI individuals [42]. Moreover, motor fatigability was similar in people with SCI with and without clinically significant perceived fatigability. We must consider that FSS is a scale used for a gross evaluation of fatigue in daily life, and thus its relationship with the hand motor functions may be tiny, even in people with cervical SCI.

### Study Limitations

This study was strengthened by a large sample of participants with SCI patients with a broad spectrum of disability levels and backgrounds. However, the cervical and thoracolumbar SCI groups were not similar in respect to severity. Patients were taking different medications that may have affected our findings.

## 5. Conclusions

The main results of this study can be summarized as follows: (1) motor fatigability induced by an isometric task is similar in able-bodied people and people with SCI; (2) motor fatigability is influenced by the maximal force (measured at the beginning of the task); and (3) the perceived fatigability and the motor fatigability are largely independent in people with SCI.

Furthermore, as many factors contribute to fatigue, future studies will be conducted to clarify which are the most relevant ones and, if possible, to determine which factors are modifiable.

## Figures and Tables

**Table 1:** Demographic characteristics of spinal cord injury participants according to the lesion level: cervical or thoracolumbar.

Variable	Thoracolumbar	Cervical	*p* Value
*N*	56	40	-
Demographic			
AGE (mean ± SD years)	47.43 ± 17.56	47.18 ± 17.89	0.945 **
SEX (Male/Female)	33/23	26/14	0.547 *
Clinical Data			
SCI etiology (Traumatic/non-Traumatic)	34/22	30/10	0.143 *
TIME SINCE INJURY (mean ± SD months)	27.37 ± 82.07	18.30 ± 39.45	0.519 **
AIS (A/B/C/D)	30/2/15/9	3/5/17/15	<0.001 ***
UEMS (median, 95% CI)	50.00	39.50 (36.15–41.54)	
LEMS (median, 95% CI)	6.00 (10.00–18.57)	31.50 (20.99–31.91)	0.001 ***
UEMS_preferred_hand (median, 95% CI)	25.00	22.00 (20.72–22.67)	
MAS (median, 95% CI)	1.00 (0.60–1.13)	1.25 (0.94–1.53)	0.038 ***
FSS (mean ± SD)	2.92 ± 1.54	3.34 ± 1.40	0.117 ***
CSF	14 (25.00%)	12 (30.00%)	0.587 *
PAIN-NRS (median, 95% CI)	3.00 (2.29–4.03)	2.50 (2.31–4.64)	0.741 ***
DEPRESSIVE MOOD	11 (19.64%)	4 (10.00%)	0.200 *

*N*: number of participants; SD: standard deviation; CI: confidence interval; SCI: spinal cord injury; AIS: American spinal injury association (ASIA) impairment scale; UEMS: upper extremity motor score; LEMS: lower extremity motor score; MAS: modified Ashworth scale; FSS: fatigue severity scale; CFS: clinically significant fatigue (FSS > 4); NRS: numeric rating scale. * = Chi-square test; ** = *t* test; *** = Mann–Whitney U test.

**Table 2:** Isometric Fatiguing Task (first 20 s) performed by control subjects and Thoracolumbar and Cervical SCI individuals.

	Controls	Thoracolumbar	Cervical	*p* value
MVC_PEAK_	28.44 ± 10.28 N	24.56 ± 10.70 N	11.04 ± 6.58 N	<0.001
MVC_MOD_	20.11 ± 7.13 N·s	16.53 ± 8.07 N·s	6.90 ± 4.91 N·s	<0.001
MVC_MOD_F	65.97 ± 19.40%	64.67 ± 20.71%	63.71 ± 23.00%	0.862 *

MVC_PEAK_: maximal voluntary contraction force peak; MVC_MOD_: modulus of the maximal voluntary contraction; MVC_MOD_F: fatigability of the modulus of the maximal voluntary contraction or fatigue state. * Very strong evidence supporting alternative hypothesis. MVC_PEAK_ and MVC_MOD_ were similar in participants with SCI with and without CSF (ANOVA: F_1,94_ = 0.640, *p* = 0.426; F_1,94_ = 0. 192, *p* = 0.663) with anecdotal evidence confirmed by Bayesian ANOVA (BF_10_ = 0.313; BF_10_ = 0.258).

## Data Availability

The raw data supporting the conclusions of this article will be made available by the authors, without undue reservation.
